# Analysis of N6-Methyladenosine Modification Patterns and Tumor Immune Microenvironment in Pancreatic Adenocarcinoma

**DOI:** 10.3389/fgene.2021.752025

**Published:** 2022-01-03

**Authors:** Yong Liu, Guangbing Li, Yang Yang, Ziwen Lu, Tao Wang, Xiaoyu Wang, Jun Liu

**Affiliations:** ^1^ Department of Liver Transplantation and Hepatobiliary Surgery, Shandong Provincial Hospital, Cheeloo College of Medicine, Shandong University, Jinan, China; ^2^ Department of Liver Transplantation and Hepatobiliary Surgery, Shandong Provincial Hospital Affiliated to Shandong First Medical University, Jinan, China

**Keywords:** N6-methyladenosine, pancreatic adenocarcinoma, tumor immune microenvironment, m6Ascore, prognosis

## Abstract

**Background:** Pancreatic adenocarcinoma (PAAD) is a rare cancer with a poor prognosis. N6-methyladenosine (m6A) is the most common mRNA modification. However, little is known about the relationship between m6A modification and the tumor immune microenvironment (TIME) in PAAD.

**Methods:** Based on 22 m6A regulators, m6A modification patterns of PAAD samples extracted from public databases were systematically evaluated and correlated with the tumor immune and prognosis characteristics. An integrated model called the “m6Ascore” was constructed, and its prognostic role was evaluated.

**Results:** Three different m6Aclusters and gene clusters were successively identified; these clusters were characterized by differences in prognosis, immune cell infiltration, and pathway signatures. The m6Ascore was constructed to quantify the m6A modifications of individual patients. Subsequent analysis revealed that m6Ascore was an independent prognostic factor of PAAD and could be a potential indicator to predict the response to immunotherapy.

**Conclusion:** This study comprehensively evaluated the features of m6A modification patterns in PAAD. m6A modification patterns play a non-negligible role in the TIME of PAAD. m6Ascore provides a more holistic understanding of m6A modification in PAAD, and will help clinicians predict the prognosis and response to immunotherapy.

## Introduction

Pancreatic adenocarcinoma (PAAD) is a rare cancer with an incidence of 12.9 cases per 100,000 person-years. Although its incidence is low, PAAD is the third and fifth most common cause of cancer death in the United States and the United Kingdom, respectively (O'Reilly et al., 2018; Owens et al., 2019). Surgical intervention is the only way to improve the chance of long-term survival (Bilimoria et al., 2007); however, most PAAD cases present with unresectable disease, which is due to either locally advanced or metastatic disease (Singhi et al., 2019). Despite the use of different therapeutic measures, the median survival time is only 6–12 months (Warshaw and Castillo, 1992; Mizrahi et al., 2020). According to recent data from the National Cancer Institute (NCI), the five-year survival rate for patients with localized PAAD is 37.4%. When distant metastases occur, the five-year survival rate drops to 2.9% (Owens et al., 2019). Since the prognosis is such poor, elucidating the genetic feature of PAAD is vital for developing valid treatments and predicting the prognosis.

In recent years, epigenetic modifications have been confirmed to be implicated in a variety of biological processes and disease progression. They mainly involved chromatin remodeling, DNA methylation, RNA modification, and histone modification (McGee and Hargreaves, 2019). More than 100 different types of post-transcriptional modifications have been confirmed in RNA (Pinello et al., 2018). Among them, N6-methyladenosine (m6A) is the most abundant mRNA modification in mammals (Dai et al., 2018). It is a reversible and complex RNA epigenetic process regulated by the interactions among m6A regulators, including “writers” (methyltransferases), “readers” (binding proteins), and “erasers” (demethylases) (Zaccara et al., 2019). m6A is involved in a variety of biological and disease processes by regulating target gene expression (Chen et al., 2019; Lan et al., 2019). Previous studies have shown that m6A is involved in cancer development and progression, including acute myeloid leukemia, breast cancer, glioblastoma, lung cancer, and hepatocellular carcinoma (Zhang et al., 2016; Li et al., 2017; Xiang et al., 2017; Dai et al., 2018). Recently, Zhou et al. (2021) constructed a model including 9 m6A regulators and found it could predict tumor aggressiveness and immune evasion in PAAD. However, the model is limited to the number of m6A regulators, while the role of them in the development and progression of PAAD depends on the interaction among multiple m6A regulators.

In this study, we systematically evaluated the features of m6A modification pattern and tumor immune microenvironment (TIME) in PAAD patients. Based on the m6A regulators and related genes, a model (termed “m6Ascore”) was constructed and then proposed as a potential molecular classification method of PAAD. The study also demonstrated that the m6Ascore could serve as a potential tool to predict the prognosis and response to immunotherapy.

## Materials and Methods

### Data Extraction and Processing

The RNA sequencing (RNA-seq) transcriptome data and corresponding clinicopathological features of PAAD samples were obtained from The Cancer Genome Atlas (TCGA) database in April 2021. Gene expression data (measured in fragments per kilobase of exon per million fragments mapped or FPKM) was transformed into transcripts per kilobase million (TPM). Simple nucleotide variation data was extracted from the TCGA database, while the copy number variation (CNV) data was obtained from the UCSC Xena Website (https://xena.ucsc.edu/). Sample differences in the tumor microenvironment (TME) were measured using Estimation of Stromal and Immune cells in Malignant Tumor tissues using Expression data (ESTIMATE) analysis with the “estimate” R package (Yoshihara et al., 2013). In addition, an eligible PAAD cohort (GSE21501) was downloaded from the Gene Expression Omnibus (GEO) database. In subsequent analysis, TCGA and GEO datasets were selected as training and validation sets, respectively.

### Acquirement of m6A Regulators and Survival Analysis

A total of 22 m6A regulators were collected from relevant studies (Li et al., 2020a; Yi et al., 2020; Zheng et al., 2021); the regulators included 7 “writers” (WTAP, METTL16, VIRMA, RBM15B, METTL3, RBM15, and ZC3H13), 13 “readers” (YTHDC1, YTHDF1, YTHDC2, YTHDF3, IGF2BP2, LRPPRC, YTHDF2, HNRNPA2B1, HNRNPC, RBMX, EIF3A, G3BP1 and FXR1), and 2 “erasers” (ALKBH5 and FTO). The prognostic role of the m6A regulators was assessed using the Kaplan-Meier (KM) diagrams and Cox proportional hazards model.

### Consensus Clustering of m6A Regulators

Based on the expression matrix of 22 m6A regulators, patients in the TCGA cohort were classified into distinct clusters according to the best cutoff using the “ConsensusClusterPlus” R package (Wilkerson and Hayes, 2010). The number of clusters and their stability were confirmed by the consensus clustering algorithm. Survival analysis between distinct clusters was measured using the KM method. Differences in the biological processes between the distinct clusters were investigated through gene set variation analysis (GSVA) (Hänzelmann et al., 2013). The “c2.cp.kegg.v7.4.symbols” gene set was obtained from the Molecular Signatures Database (MSigDB). Adjusted *p*-value<0.05 was considered statistically significant.

### Comparison of the TIME Between Distinct m6Aclusters

The single-sample Gene Set Enrichment Analysis (ssGSEA) algorithm was used to quantify the relative abundance of various immune cell subtypes in PAAD samples (Charoentong et al., 2017). Through enrichment score calculated by ssGSEA, the relative abundance of each immune cell type was represented in each sample. ESTIMATE analysis was performed to compare the differences in the TME with the “estimate” R package (Yoshihara et al., 2013). Furthermore, differences in the TIME and the expression of targeted immune checkpoint molecules between the distinct clusters were compared using the “limma” R package.

### Identification of Prognosis-Related DEGs Between m6Aclusters

Principal component analysis (PCA) was used to test whether m6A regulators could separate distinct m6A modification patterns. Differentially expressed genes (DEGs) among the m6Aclusters were identified using the empirical Bayesian approach with the “limma” R package. The significance criterion of DEGs was set as *p*-value < 0.0001. The Kyoto Encyclopedia of Genes and Genomes (KEGG) pathway analysis and Gene Ontology (GO) biological processes analysis were performed to investigate the pathway signatures of the DEGs. A critical value of adjusted *p*-value = 0.05 was selected as the filter criteria. After identifying the DEGs, prognosis-related genes were filtrated from the DEGs by univariate Cox regression analysis. The significance criterion was set as *p*-value < 0.001.

### Consensus Clustering of Prognosis-Related DEGs

Based on the expression of prognosis-related DEGs, samples in the TCGA cohort were classified into different subtypes according to the best cutoff using the “ConsensusClusterPlus” R package. The KM method was used to perform survival analysis between different subtypes. A heatmap revealed the expression of prognosis-related DEGs between different subtypes using the “pheatmap” R package. Furthermore, differences in immune cell infiltration, ESTIMATE score and the expression of targeted immune checkpoint molecules were compared with the “limma” R package.

### Construction of the m6Ascore Model

Based on the expression of prognosis-related DEGs, PCA was used to score the samples in the TCGA and GEO cohorts. Principal components 1 and 2 were used to act as signature scores. The m6Ascore was defined using a method similar to Genomic Grade Index (GGI) (Sotiriou et al., 2006; Zeng et al., 2019):

m6Ascore=∑(PC1 i+PC2 i)

Where i is the expression of overlapping genes with significant prognosis-related DEGs the m6Aclusters.

According to the score, samples were divided into low- and high-m6Ascore groups. We then compared the biological differences between the low- and high-m6Ascore groups, including (O'Reilly et al., 2018) survival analysis (Owens et al., 2019), immuno-correlation analysis (Bilimoria et al., 2007), clinical-correlation analysis (Singhi et al., 2019), tumor mutation burden (TMB) (Mizrahi et al., 2020), targeted immune checkpoint molecules.

### Statistical Analysis

All statistical analyses in the study were performed using the R software (version 4.0.5). The Kruskal-Wallis test was used to perform difference comparison on three or more groups (Hazra and Gogtay, 2016). Continuous variables were dichotomized for patient survival using the optimal cutoff values determined by “survminer” R package. The KM and log-rank tests were used to evaluate the survival difference among different clusters. The CNV landscape of 22 m6A regulators in 23 pairs of chromosomes was plotted using the “RCircos” R package. The receiver operating characteristic (ROC) curves (R package “timeROC”) and the area under the curve (AUC) values were used to evaluate the prognostic ability of the m6Ascore for 1-, 2-, 3-, and 4-year overall survival (OS) (Blanche et al., 2013). Univariate and multivariate independent prognostic analyses were performed to assess whether the model was an independent prognostic factor of PAAD. All statistical *p* values were two-sided, with *p* < 0.05 deemed statistically significant.

## Results

### Expression Variation of the m6A Regulators in PAAD

**FIGURE 1:**
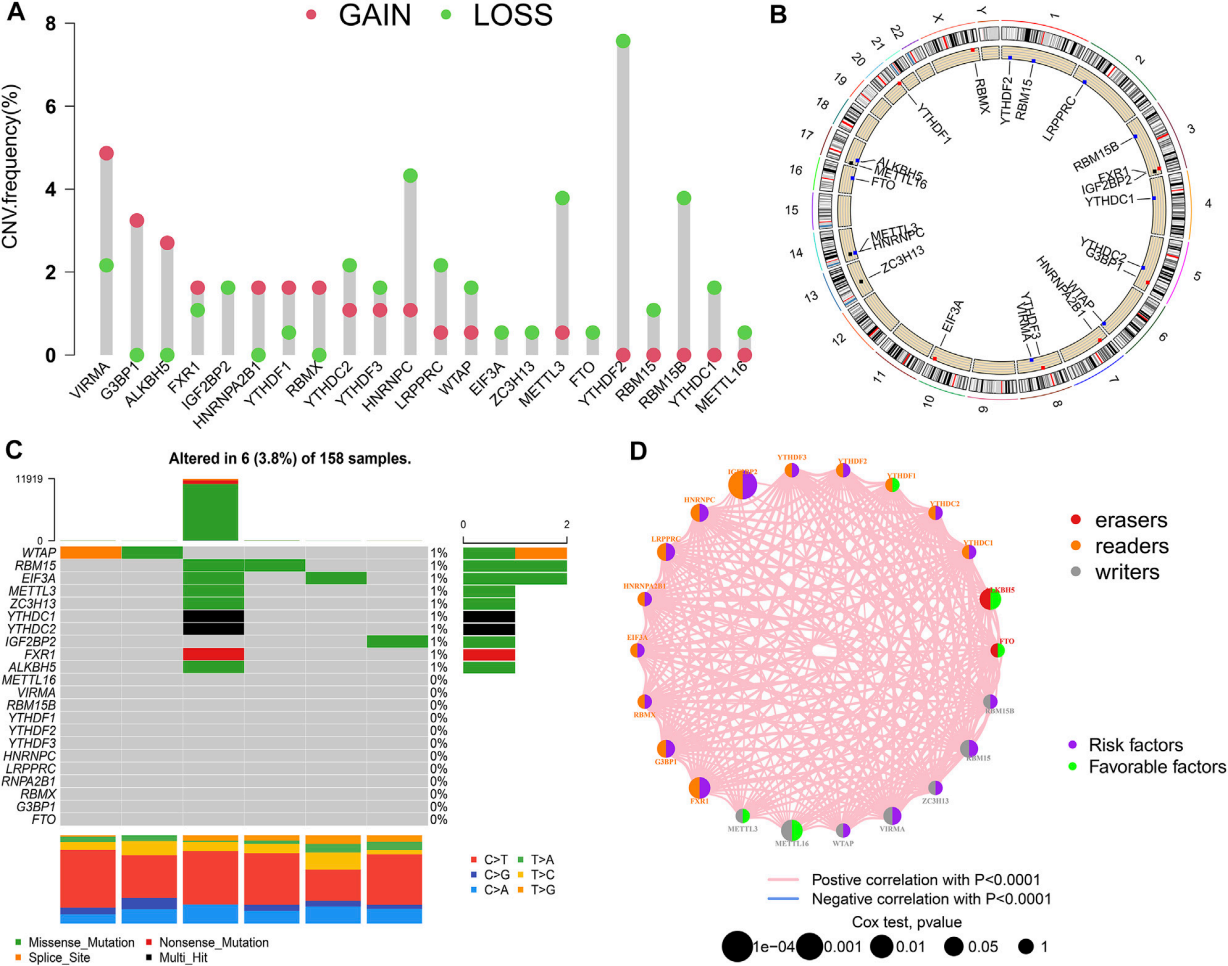
m6A modification pattern in PAAD. **(A)** The CNV alteration frequency of 22 m6A regulators in PAAD; **(B)** The CNV landscape of m6A regulators in 23 pairs of chromosomes; **(C)** The mutation frequency of m6A regulators in PAAD; **(D)** Interaction of m6A regulators in PAAD.

A total of 22 m6A regulators (7 “writers,” 13 “readers,” and 2 “erasers”) were collected in this study. CNVs and somatic mutations were integrated to explore the prevalence of m6A regulator variations in PAAD. The CNV incidence of 22 m6A regulators are shown in Figure 1A. Most regulators focused on the deletion of copy number, while VIRMA, G3BP1, and other five m6A regulators had a prevalent frequency of CNV amplification. The CNV landscape of m6A regulators in 23 pairs of chromosomes are shown in Figure 1B. The overall average mutation frequency of m6A regulators was low, with only 6 (3.8%) of 158 samples having m6A regulator mutation (Figure 1C).

The prognostic value of the m6A regulators was evaluated by KM method and univariate Cox regression analysis (Supplementary Figure S1; Supplementary Table S1). The results showed that most m6A regulators were associated with survival. The network of m6A regulators comprehensively demonstrated the m6A regulators’ interactions, connection, and prognostic significance for PAAD patients (Figure 1D). The results showed that there was a distinct positive correlation between each other. Most regulators, such as IGF2BP2 and HNRNPC, presented tumorigenic characteristics, with higher gene expression levels correlating with poor prognosis. Conversely, several m6A regulators, such as ALKBH5 and METTL16, presented tumor-suppressing characteristics, with higher gene expression levels relating to favorable prognosis. These results suggested that the interrelations among regulators may have important effects on the development and progression of PAAD.

### m6A Modification Patterns Mediated by m6A Regulators

**FIGURE 2:**
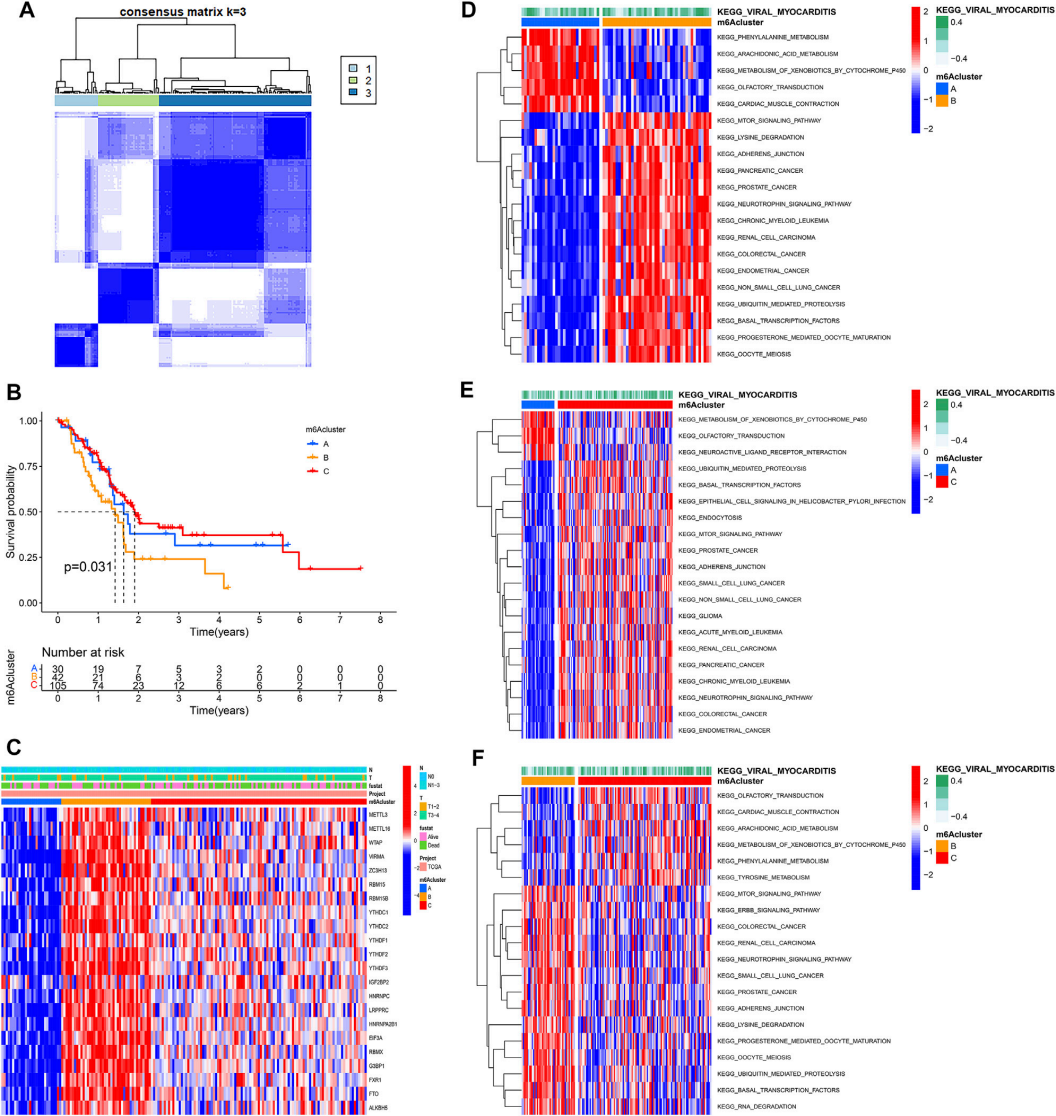
Molecular characteristics of m6Aclusters. **(A)** Consensus clustering matrix for k = 3; **(B)** KM analysis of patients in distinct m6Aclusters; **(C)** The heatmap depicted the expression levels of m6A regulators in distinct m6A clusters; **(D)** GSVA enrichment analysis between m6Acluster A and m6Acluster B; **(E)** GSVA enrichment analysis between m6Acluster A and m6Acluster C; **(F)** GSVA enrichment analysis between m6Acluster B and m6Acluster C.

Based on the expression of 22 m6A regulators, model-based clustering was performed to classify PAAD patients using the “ConsensusClusterPlus” R package. Through unsupervised clustering, three different m6A modification patterns were uncovered ultimately (identified as m6Aclusters A-C), including 30 samples in cluster A, 42 samples in cluster B, and 105 samples in cluster C (Figure 2A). Prognostic analysis showed there was a survival disadvantage in m6Acluster B (Figure 2B). The heatmap showed m6Acluster A presented significantly low expression levels of all m6A regulators, while m6Acluster B was characterized by the high expression levels of all m6A regulators (Figure 2C). Moreover, GSVA showed different biological behaviors between the m6Aclusters (Figures 2D–F). The results suggested that different m6A modifications had significant correlation with biological behaviors and prognosis of PAAD.

### Tumor Immune Landscape in Distinct m6Aclusters

**FIGURE 3:**
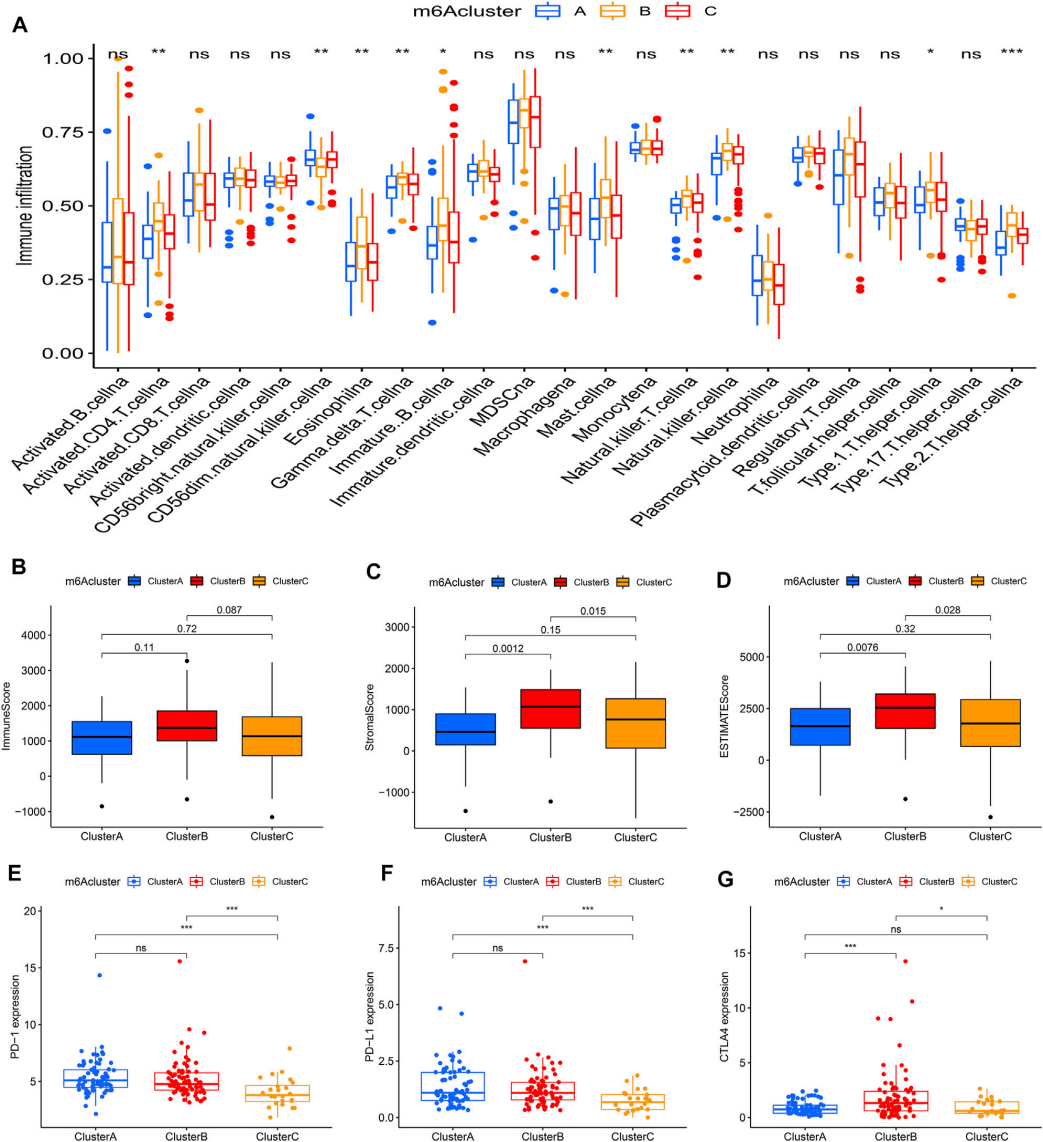
Tumor immune landscapes in distinct m6Aclusters. **(A)** ssGSEA of patients in distinct m6Aclusters, the asterisks represented the statistical *p* value (*p < 0.05; **p < 0.01; ***p < 0.001); **(B)** Immune score of patients in distinct m6Aclusters; **(C)** Stromal score of patients in distinct m6Aclusters; **(D)** ESTIMATE score of patients in distinct m6Aclusters; **(E)** PD-1 expression of patients in distinct m6Aclusters (*p < 0.05; **p < 0.01; ***p < 0.001); **(F)** PD-L1 expression of patients in distinct m6Aclusters (*p < 0.05; **p < 0.01; ***p < 0.001); **(G)** CTLA4 expression of patients in distinct m6Aclusters (*p < 0.05; **p < 0.01; ***p < 0.001).

Using ssGSEA, the study analyzed 23 different immune cell types in the m6Aclusters. The result revealed that m6Acluster B, which had a poor prognosis, was enriched in activated NK cells, mast cells, and T helper type 2 (Th2) cells. However, the abundance of CD56dim NK cells was enriched in m6Aclusters A and C (Figure 3A). These results indicated that m6A modification was associated with the infiltration of specific immune cell types and influenced the response to immunotherapy. In addition, the results of the ESTIMATE algorithm revealed that the stromal and ESTIMATE scores (*p* < 0.05) were higher in cluster B than in clusters A and C (Figures 3B–D). Combined with the heatmap, the study found that the expression level of m6A regulators showed a similar trend with the ESTIMATE score. Characterized by the higher expression levels of m6A regulators, m6Acluster B also had a higher ESTIMATE score. The results suggested that m6A regulators may play an important role in the regulation of the TME, thus affecting tumor progression and survival. Furthermore, the expression of targeted immune checkpoint molecules was different between the distinct clusters. As shown in the boxplots, the expression of the CTLA-4 gene was markedly high in m6Acluster B and the expression levels of the PD-1 and PD-L1 genes were markedly low in m6Acluster C (Figures 3E–G).

### Generation of m6A Gene Clusters

**FIGURE 4:**
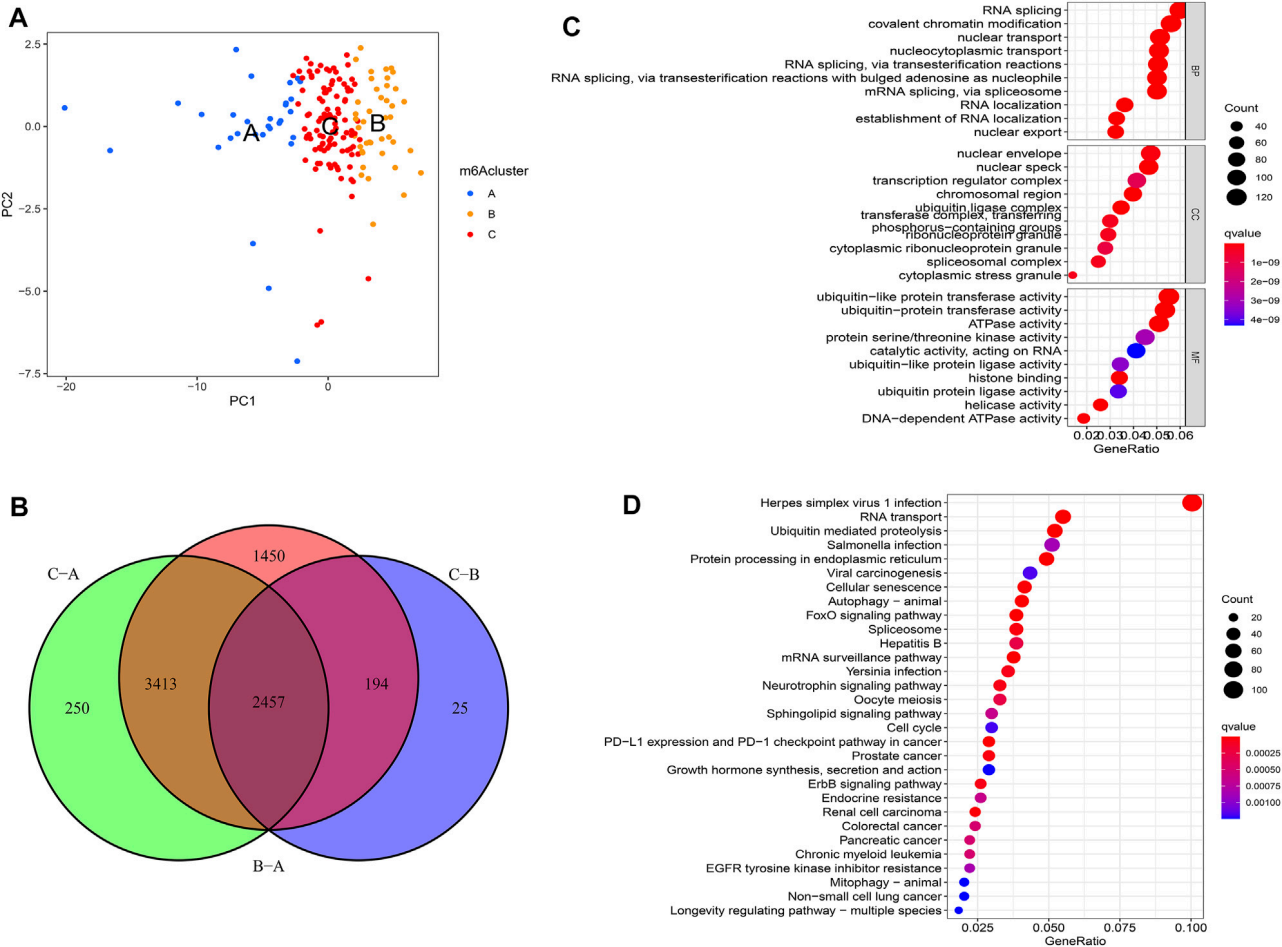
Molecular characteristics of differentially expressed genes (DEGs) among m6Aclusters. **(A)** PCA among distinct m6Aclusters; **(B)** The DEGs extracted between three m6A clusters; **(C)** GO enrichment analysis for the DEGs; **(D)** KEGG pathway analysis for the DEGs.

PCA showed that m6A regulators could separate distinct m6A modification patterns perfectly (Figure 4A). To further investigate the potential biological behavior of each m6Acluster, a total of 2457 DEGs among three m6Aclusters were extracted eventually (Figure 4B). Similarly, the “clusterProfiler” R package was used to implement GO enrichment analysis and KEGG pathway analysis for the DEGs. The results showed that the DEGs were enriched in biological processes related to tumorigenesis and tumor progression, such as FoxO signaling pathway and ErbB signaling pathway (Figures 4C,D). The results revealed that m6A modification played a significant role in the tumorigenesis and tumor progression of PAAD.

**FIGURE 5:**
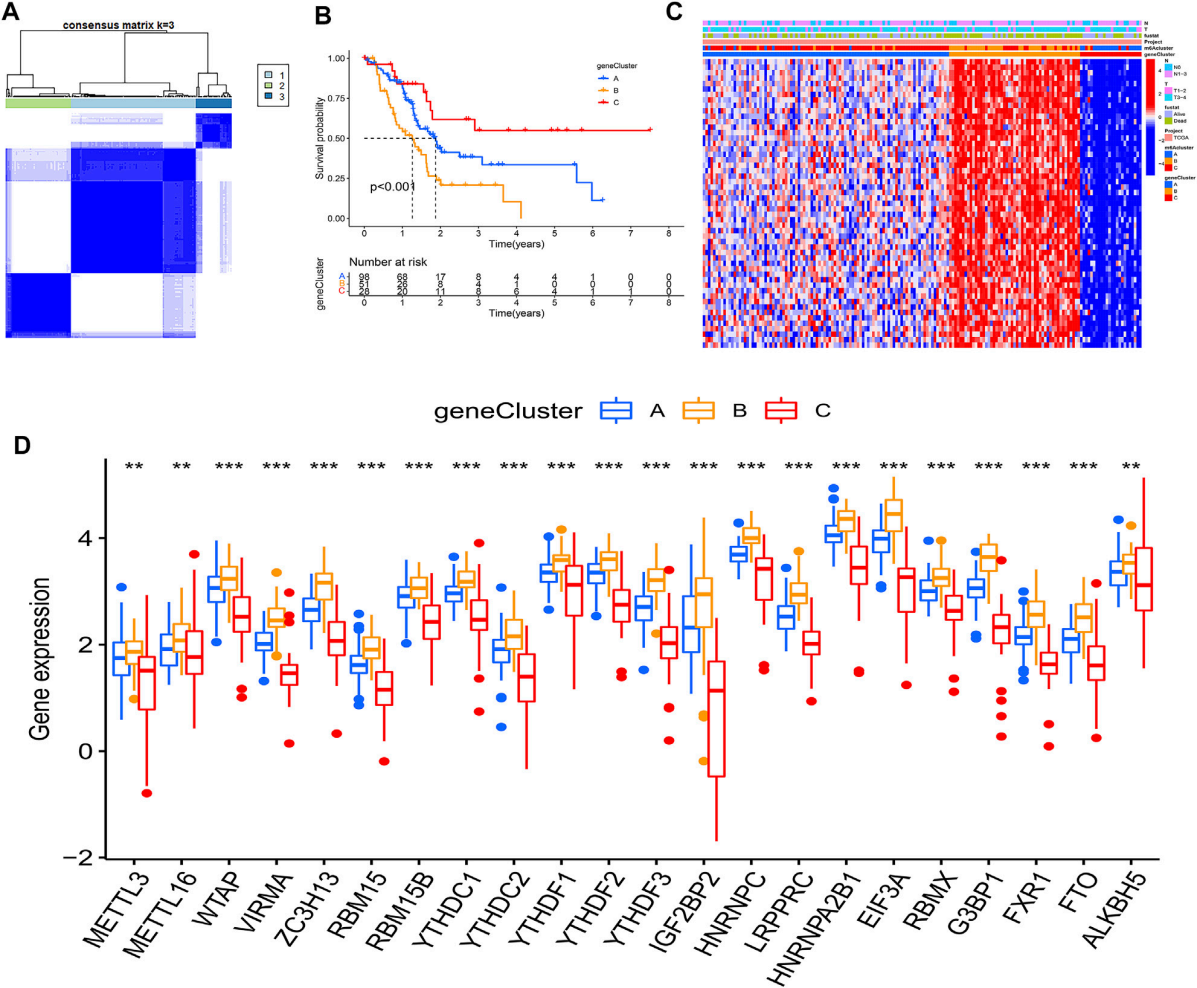
Molecular characteristics of gene clusters. **(A)** Consensus clustering matrix for k = 3; **(B)** KM analysis of patients in distinct gene clusters; **(C)** The heatmap depicted the expression levels of prognosis-related DEGs in distinct gene clusters; **(D)** Expression of m6A regulators between distinct gene clusters, the asterisks represented the statistical *p* value (*p < 0.05; **p < 0.01; ***p < 0.001).

**FIGURE 6:**
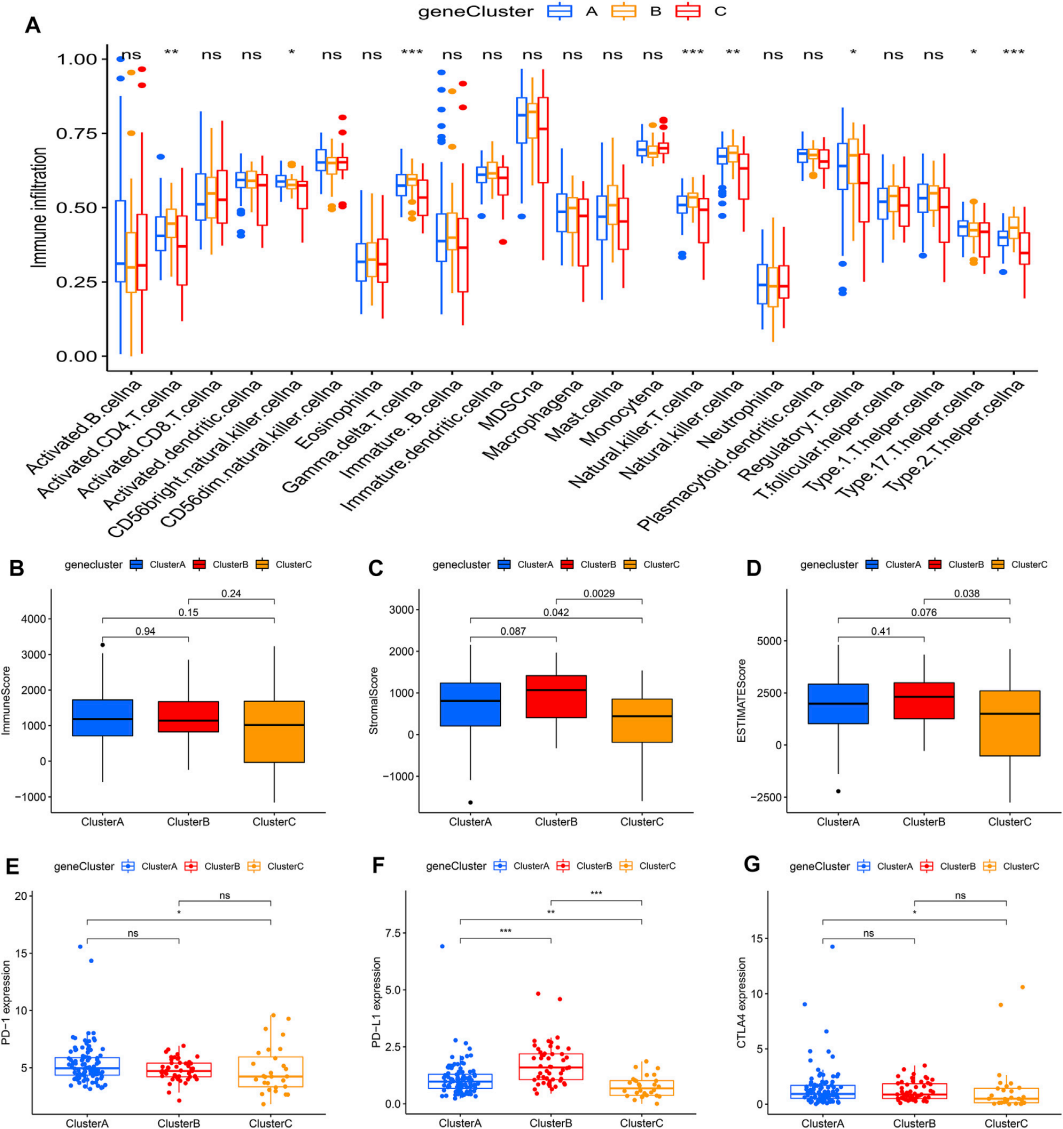
Tumor immune landscapes in distinct gene clusters. **(A)** ssGSEA of patients in distinct gene clusters, the asterisks represented the statistical *p* value (*p < 0.05; **p < 0.01; ***p < 0.001); **(B)** Immune score of patients in distinct gene clusters; **(C)** Stromal score of patients in distinct gene clusters; **(D)** ESTIMATE score of patients in distinct gene clusters; **(E)** PD-1 expression of patients in distinct gene clusters (*p < 0.05; **p < 0.01; ***p < 0.001); **(F)** PD-L1 expression of patients in distinct gene clusters (*p < 0.05; **p < 0.01; ***p < 0.001); **(G)** CTLA4 expression of patients in distinct gene clusters (*p < 0.05; **p < 0.01; ***p < 0.001).

To further investigate this regulation mechanism, univariate Cox regression analysis was performed to extract prognosis-related genes among the DEGs (Supplementary Table S2). Based on the 53 prognosis-related DEGs obtained, patients were divided into three genomic subtypes through unsupervised clustering analyses (gene clusters A-C) (Figure 5A). Survival analysis showed that patients in gene cluster B had a worse outcome, while these in gene cluster C showed a prominent survival advantage (Figure 5B). The heatmap revealed that gene clusters A-C were characterized by different signature genes (Figure 5C). Prognosis-related DEGs were overexpressed in gene cluster B, and under-expressed in gene cluster C. Moreover, gene cluster B showed higher expression levels of all m6A regulators, while gene cluster C had lower expression levels of all m6A regulators (Figures 5D; Supplementary Figure S2A). Similarly, GSVA showed different biological behaviors between three gene clusters (Supplementary Figures S2B–D). In addition, ssGSVA showed gene cluster B had a high abundance of Th2 cells and NK cells (Figure 6A). The stromal and ESTIMATE scores (*p* < 0.05) were higher in cluster B compared to cluster C (Figures 6B–D). The results further demonstrated that m6A modification patterns were tightly associated with the TME of PAAD. Similarly, the expression of targeted immune checkpoint molecules was different between the distinct gene clusters (Figures 6E–G).

### Construction of the m6Ascore Model

**FIGURE 7:**
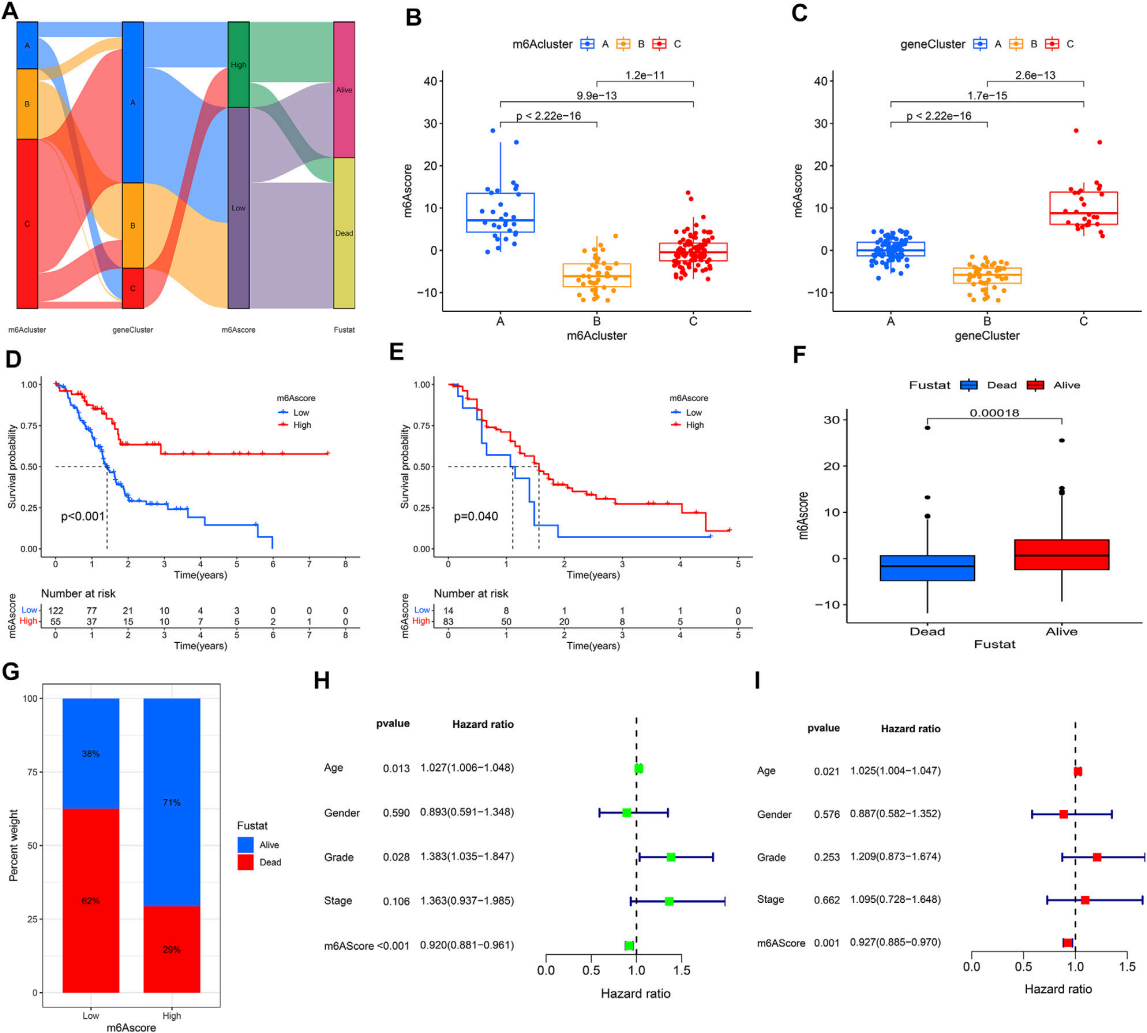
Construction of m6Ascore model. **(A)** Alluvial diagram showed the changes of m6Aclusters, gene clusters, and m6Ascore; **(B)** Differences in m6Ascore between three m6Aclusters; **(C)** Differences in m6Ascore between three gene clusters; **(D)** KM analysis of patients in the high- and low-m6Ascore groups (TCGA cohort); **(E)** KM analysis of patients in the high- and low-m6Ascore groups (GEO cohort); **(F,G)** Clinical correlation analysis; **(H)** Univariate independent prognostic analysis; **(I)** Multivariate independent prognostic analysis.

Considering the individual heterogeneity and complexity of m6A modification in PAAD, the study used PCA to quantify the m6A modification pattern of samples based on the prognosis-related DEGs and then divided them into low- and high-m6Ascore groups. The alluvial diagram was used to visualize the attribute changes of individual samples, which showed that m6Acluster B had a high proportion of gene cluster B and was linked to a low m6Ascore (Figure 7A). Furthermore, Kruskal-Wallis test indicated a difference in m6Ascore among the m6Aclusters. The results showed that m6Acluster B had the lowest median score, while m6Acluster A had the highest median score (Figure 7B). The similar results were obtained when analyzing the correlation between m6Ascore and gene clusters. Gene cluster B had the lowest median score, while gene cluster C had the highest median score (Figure 7C).

**FIGURE 8:**
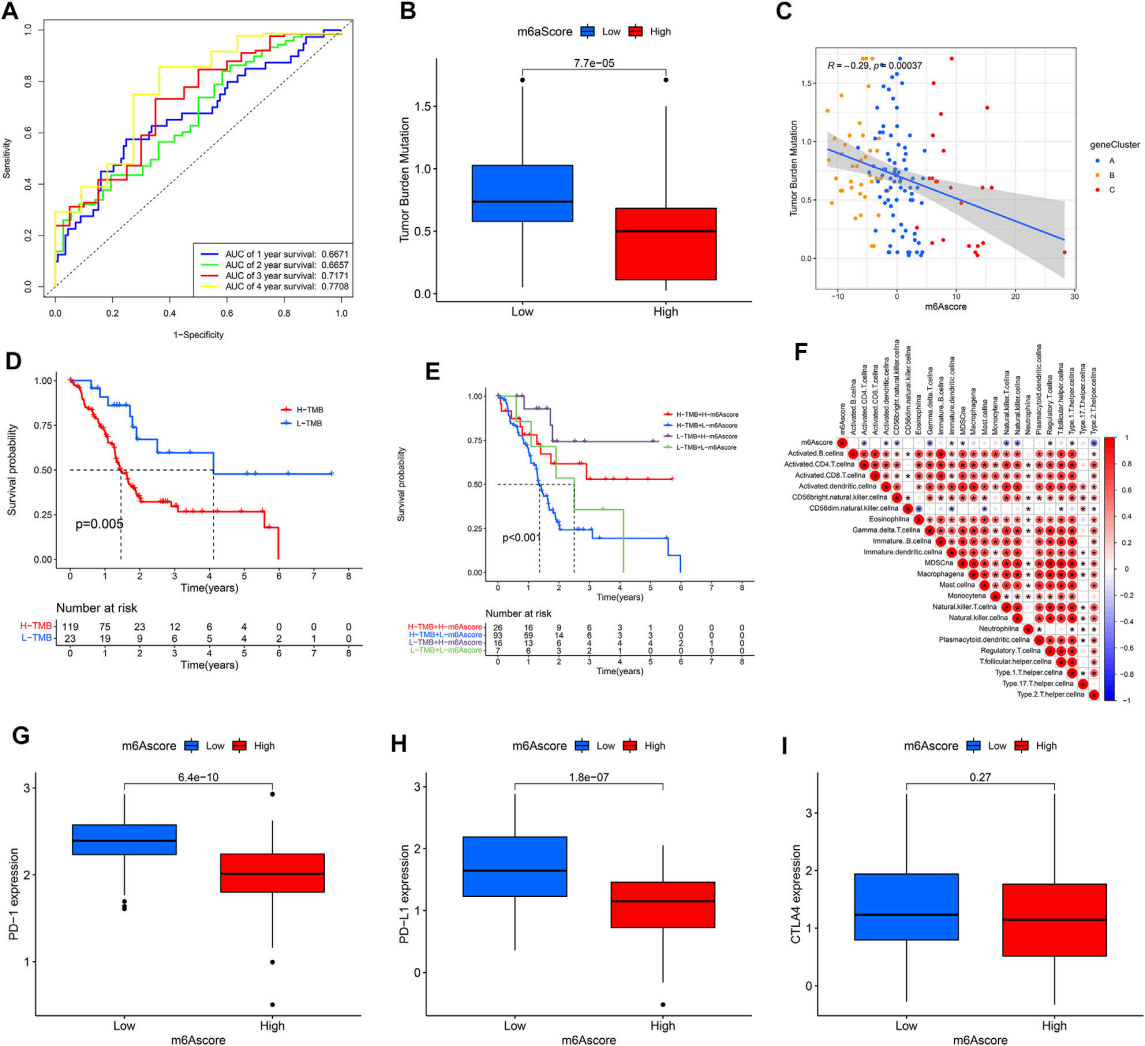
Clinical value of m6Ascore model. **(A)** Receiver operating characteristic (ROC) curves of m6Ascore for predicting the 1/2/3/4-year survival; **(B,C)** tumor mutation burden (TMB) correlation analysis; **(D)** KM analysis of patients with distinct TMB; **(E)** KM analysis of patients with distinct TMB and m6Ascore; **(F)** Immuno-correlation analysis; **(G)** PD1 expression of patients in distinct m6Ascore groups; **(H)** PD-L1 expression of patients in distinct m6Ascore groups; **(I)** CTLA4 expression of patients in distinct m6Ascore groups.

Patients in the high m6Ascore group demonstrated a prominent survival benefit in both the TCGA cohort and GEO cohort (GSE21501) (Figures 7D,E). Moreover, there was a negative correlation between m6Ascore and survival state. The low m6Ascore group had a high proportion of patients in dead (Figures 7F,G). As shown in Figure 7H, m6Ascore was markedly related to OS (hazard ratio (HR): 0.920, 95% confidence interval (CI): 0.881–0.961, *p* < 0.001). In addition, a multivariate Cox regression model including age, gender, tumor grade, m6Ascore, and tumor stage confirmed that m6Ascore was an independent prognostic factor of PAAD (HR: 0.927, 95% CI: 0.885–0.970, *p* = 0.001) (Figure 7G). ROC analysis revealed that m6Ascore had an acceptable prognostic value for PAAD patients (1-year AUC = 0.6671, 2-year AUC = 0.6657, 3-year AUC = 0.7171, 4-year AUC = 0.7708; respectively) (Figure 8A). These results indicated that m6Ascore had a robust and stable OS-predictive ability for PAAD.

As shown in Figures 8B,C, there was a negative correlation between the m6Ascore and the TMB. Patients with high TMB had a low m6Ascore. Survival analysis revealed that patients with high TMB had a poor prognosis; and the similar result was obtained from patients who had high TMB and low m6Ascore (Figures 8D,E). Of note, there were negative correlations between m6Ascore and some infiltrated immune cells, including activated Th2 cells, regulatory T cells, and NK cells et al. In addition, the expression levels of the PD-1and PD-L1 genes were higher in patients with low m6Ascore, while there was no difference in the CTLA4 gene expression (Figures 8G–I).

## Discussion

PAAD is a highly lethal malignancy, and its therapy remains a formidable challenge (Leinwand and Miller, 2020). Clinical efforts to use immune therapy have been shown to be largely ineffective for PAAD patients (Feng et al., 2017). With such a poor outcome, it is urgent to investigate the genetic features of PAAD and identify novel therapeutic strategies to improve its prognosis. m6A is regarded as the most pervasive, abundant, and conserved internal modification in RNAs, including mRNA, non-coding RNA, and ribosomal RNA (Fazi and Fatica, 2019). Considerable evidence indicated that the collaboration between m6A regulators played an important role in tumorigenesis, tumor progression, and immune response (Chang et al., 2019; Li et al., 2020b; Zhang et al., 2020). Previous studies have demonstrated that m6A modification was associated with the occurrence and development of PAAD. WTAP could promote tumor metastasis via stabilizing Fak mRNA and would result in a poor prognosis (Li et al., 2021a). Meanwhile, Xia et al. (2019) revealed that METTL3 promoted tumor cell proliferation and invasion, and could be a treatment target. In addition, it was found that ALKBH5 could prevent tumor progression by regulating the posttranscriptional activation of PER1 (Guo et al., 2020). However, almost all studies have focused on single m6A regulator only. How m6A modification pattern mediate the TIME and tumor survival in PAAD remains unknown.

In this study, based on data from public databases, we comprehensively and systematically profiled the m6A modification patterns in PAAD patients. Using unsupervised clustering analyses, three m6Aclusters and gene clusters have been successively identified. A series of biological analyses were performed to explore the relationship between the m6A-related genes and the TIME in PAAD. In addition, a model called “m6Ascore” was constructed to quantify the m6A modifications of individual patients. Subsequent analysis revealed that m6Ascore was an independent prognostic factor of PAAD and could be a potential indicator to predict response to immunotherapy.

As discovered by the Human Genome Project, many genetic regions display a variation in the number of copies. These genetic variants are termed CNVs and are defined as a DNA segment that is 1 kb or larger and present at variable copy number in comparison with a reference genome (Feuk et al., 2006). A CNV can be simple in structure, such as tandem duplication, or may involve complex gains or losses of homologous sequences at multiple sites in the genome (Redon et al., 2006). CNVs influence gene expression and phenotypic variation by disrupting genes and altering gene dosage (McCarroll et al., 2006). Previous studies have found the presence of CNVs in the human genome and their associations with cancers (Cheng et al., 2016; Verma and Sharma, 2018; Santarpia et al., 2016). The larger a CNV, the more likely it is to be associated with disease; however, the phenotypic effects are often unclear and unpredictable. In our study, the CNV incidence of YTHDF2 was higher than those of YTHDF1 and YTHDF3. However, there was no difference in the expression of these genes between tumor and normal tissues (Supplementary Figure S3). In addition, the prognostic value of YTHDF1-3 was evaluated by KM method and univariate Cox regression analysis. The results showed that all of them were not associated with survival. Of note, Chen et al. (2017) found that YTHDF2 is up-regulated in PAAD and associated with the poor stage of patients. The reason for the different results may be that in our study, there were only a few normal samples and high-stage tumor samples. Furthermore, the tumor samples in our study contained different clinicopathological features, including age, gender, tumor grade. In addition, YTH-family genes play different role in PAAD. For instance, YTHDF2 orchestrated two cellular processes via TGF-β/Smad signaling pathway: promoted proliferation and inhibited migration and invasion in pancreatic cancer cells (Chen et al., 2017). Thus, further research with more samples was needed to explore this issue.

Since there were distinct correlations between the m6A regulators, patients were stratified into three m6Aclusters, which were different in prognosis, immune cell infiltration, and pathway signatures. The study found that the expression levels of m6A regulators were associated with the prognosis of PAAD. Patients with the high expression levels of m6A regulators had a poor prognosis. Of note, there was a positive correlation between m6A regulators and the ESTIMATE score. m6Acluster B, which had a high ESTIMATE score, was characterized by the high expression levels of m6A regulators. ESTIMATE score was used to assess the level of infiltrating stromal and immune cells and infer tumor purity in tumor tissue, with a high ESTIMATE score indicating low tumor purity. Relevant studies have indicated that low tumor purity was related to an unfavorable prognosis in glioma and colon cancer, which was similar to our finding (Zhang et al., 2017; Mao et al., 2018). Moreover, there was significant difference in immune cell infiltration between the distinct clusters. m6Acluster B and gene cluster B, which had a poor prognosis, were characterized by the high infiltration level of NK cells and Th2 cells. Relevant research showed that a high number of NK cells was correlated with a poor prognosis in PAAD. This may be due to tumor cells affected the activation of NK cells by inhibiting IL-2, IFN-γ, and TNF-α secretion, thus rendering them inept (Yang et al., 2018). In addition, Th2 cells were correlated with cancer-associated fibroblast thymic stromal lymphopoietin and a high abundance of them could reduce survival in PAAD (De Monte et al., 2011).

TME is a complex system with multiple components, including immune cells and non-immune cells, that plays a crucial role in cancer development and progression (Ocaña et al., 2019). Accumulating studies have suggested that m6A modification played an important role in TME. For instance, large abnormalities of m6A mRNA were found in immune cells such as Dendritic cells (DCs). In this context, the altered m6A mRNA powerfully contributed to immune disorders and tumor escape, partially through the inhibition of immune cell function and migration (Li et al., 2021b). YTHDF1 was confirmed to induce the expression of lysosomal proteases by recognizing their m6A-marked mRNAs and increasing translation efficiency, which caused DCs to be unable constantly cross-present engulfed tumor neoantigens and then impeded the antigen-specific activation of CD8 + T cells (Han et al., 2019). Moreover, METTL14 and WTAP were confirmed to participate in the regulation of vascular endothelial cells (VECs) functions (He et al., 2019; Wang et al., 2020). In addition, increasing evidence has revealed that m6A methylation regulated TME remodeling in tumor metastasis, including gastric cancer, lung cancer and ovarian cancer (Hua et al., 2018; Yue et al., 2019; Wanna-Udom et al., 2020). For instance, METTL3-mediated m6A controlled TGF-β-induced epithelial-mesenehymal transition (EMT) in cancer cells, and obviously suppressed lung metastasis *in vivo* in response to METTL3 deficiency (Yue et al., 2019).

Considering the complex reciprocal regulatory relation between the m6A-related genes, it is necessary to accurately evaluate the m6A modification patterns of individual PAAD patients. In this study, a model (called “m6Ascore”) was constructed. Based on m6Ascore, patients were divided into low- and high-m6Ascore groups. Patients in the low m6Ascore group demonstrated a poor prognosis. Integrated analyses demonstrated that m6Ascore was a robust and independent prognostic factor of PAAD. Meanwhile, m6Ascore was negatively correlated with the TMB and patients with a high TMB had a low m6Ascore. TMB represents the somatic coding errors such as base substitutions, insertions or deletion (Chan et al., 2019). A high TMB was found to promote immune cell infiltration and antigen formation, which could strengthen the immune response and improve immunotherapy efficacy in multiple cancers (Miao et al., 2018; Chan et al., 2019). In addition, the expression of targeted immune checkpoint molecules PD-1 and PD-L1 was high in the low m6Ascore group. Tumors often up-regulate immune checkpoints to avoid being detected and killed by the host immune system. Activation of checkpoint cascades such as those controlled by PD-1 or PD-L1 will result in inactivation of tumor-specific T cells and immune evasion (Iwai et al., 2002; Dunn et al., 2004; Brown et al., 2010). Treatment with anti-PD-1 and anti-PD-L1 could reinvigorate T cells and allow the adaptive immune system to target tumor cells (Pardoll, 2012). Previous studies have shown that the expression of PD-1 and PD-L1 could be as predictive biomarkers for immunotherapy response (Ferris et al., 2016; Herbst et al., 2016; Muro et al., 2016; Ott et al., 2017). Therefore, based on the close relationship between m6Ascore, TMB and the significant difference in the expression of targeted immune checkpoint molecules, m6Ascore could be identified as a potential and effective indicator to predict the response to immunotherapy.

Some limitations of this study have been observed. First, immune cell infiltration was assessed based on algorithms owing to technical limitations. Second, the regulatory mechanism of m6A regulators in TIME was not explored exhaustively, which needed further investigation. Last, there was no clinical cohort to verify the predictive value of m6Ascore in PAAD, thus, further research based on large cohort prospective clinical trials was needed.

In conclusion, this study comprehensively identified and systematically profiled the genetic features of m6A-related regulators in PAAD. Distinct m6A modification patterns contacted with different prognoses, immune cell infiltrations, and pathway signatures. The study also constructed a m6Ascore model, which was a potential therapeutic signature for PAAD. This study will help clinicians identify potential indicators of PAAD to improve the poor prognosis of this disease.

## Data Availability

The datasets presented in this study can be found in online repositories. The names of the repository/repositories and accession number(s) can be found in the article/Supplementary Material.

## Supplementary Material

Supplementary Figure 1 Survival analysis of m6A regulators. **(A)** KM analysis of patients with distinct ALKBH5 expression; **(B)** KM analysis of patients with distinct EIF3A expression; **(C)** KM analysis of patients with distinct FTO expression; **(D)** KM analysis of patients with distinct FXR1 expression; **(E)** KM analysis of patients with distinct G3BP1 expression; **(F)** KM analysis of patients with distinct HNRNPA2B1 expression; **(G)** KM analysis of patients with distinct HNRNPC expression; **(H)** KM analysis of patients with distinct IGF2BP2 expression; **(I)** KM analysis of patients with distinct LRPPRC expression; **(J)** KM analysis of patients with distinct METTL3 expression; **(K)** KM analysis of patients with distinct METTL16 expression; **(L)** KM analysis of patients with distinct RBM15 expression; **(M)** KM analysis of patients with distinct VIRMA expression; **(N)** KM analysis of patients with distinct YTHDF3 expression; **(O)** KM analysis of patients with distinct ZC3H13 expression.

Supplementary Figure 2 Molecular characteristics of gene clusters. **(A)** The heatmap depicted the expression levels of m6A regulators in distinct gene clusters; **(B)** GSVA enrichment analysis between gene cluster A and gene cluster B; **(C)** GSVA enrichment analysis between gene cluster A and gene cluster C; **(D)** GSVA enrichment analysis between gene cluster B and gene cluster C.

Supplementary Figure 3 Molecular characteristics of YTH-family genes. **(A–C)** The expression differences of YTHDF1-3 between tumor and normal tissues; **(D–F)** The expression differences of YTHDF1-3 between the different tumor stage; **(G)** KM analysis of patients with distinct YTHDF1-3 expression.
