# Supplementary material for: Analysis of N6-Methyladenosine Modification Patterns and Tumor Immune Microenvironment in Pancreatic Adenocarcinoma
Source: Front Genet. 2022 Jan 3;12:752025. doi: 10.3389/fgene.2021.752025 (PMC8762218; doi:10.3389/fgene.2021.752025)
Supplement: Supplementary file 1 [file Table1.DOCX]

Table S1 Univariate Cox regression analysis of m6A regulators.

| ID | HR | HR.95L | HR.95H | pvalue | km |
| --- | --- | --- | --- | --- | --- |
| METTL3 | 0.767191 | 0.564577 | 1.042518 | 0.090297 | 0.00815 |
| METTL16 | 0.549308 | 0.365753 | 0.824982 | 0.003888 | 0.006195 |
| WTAP | 1.025117 | 0.657564 | 1.598118 | 0.912806 | 0.268018 |
| VIRMA | 1.684141 | 1.043902 | 2.717049 | 0.032677 | 0.00213 |
| ZC3H13 | 1.171399 | 0.772242 | 1.776871 | 0.456773 | 0.020114 |
| RBM15 | 1.706851 | 1.015925 | 2.867671 | 0.04342 | 0.010677 |
| RBM15B | 1.119252 | 0.647614 | 1.93437 | 0.686516 | 0.084238 |
| YTHDC1 | 1.00624 | 0.618236 | 1.637754 | 0.980031 | 0.172361 |
| YTHDC2 | 1.008367 | 0.682091 | 1.490716 | 0.96668 | 0.295201 |
| YTHDF1 | 0.827907 | 0.499478 | 1.372294 | 0.463878 | 0.052318 |
| YTHDF2 | 1.438784 | 0.856289 | 2.417525 | 0.169442 | 0.105933 |
| YTHDF3 | 1.250773 | 0.856746 | 1.826016 | 0.246426 | 0.01313 |
| IGF2BP2 | 1.586611 | 1.270681 | 1.981091 | 4.61E-05 | 0.000375 |
| HNRNPC | 2.057577 | 1.087374 | 3.89344 | 0.026596 | 0.009585 |
| LRPPRC | 1.888246 | 1.126421 | 3.165311 | 0.015882 | 7.91E-05 |
| HNRNPA2B1 | 1.379083 | 0.850622 | 2.235857 | 0.192327 | 0.031475 |
| EIF3A | 1.413232 | 0.982265 | 2.033284 | 0.062385 | 0.001097 |
| RBMX | 1.106378 | 0.63362 | 1.931874 | 0.72224 | 0.225718 |
| G3BP1 | 1.677345 | 1.123054 | 2.505208 | 0.011505 | 0.0007 |
| FXR1 | 2.146404 | 1.298164 | 3.548896 | 0.00291 | 8.68E-05 |
| FTO | 0.988527 | 0.643272 | 1.519087 | 0.95802 | 0.036215 |
| ALKBH5 | 0.583999 | 0.388254 | 0.878431 | 0.009815 | 0.008001 |

Abbreviations: HR: hazard ratio.
