# Supplementary figures and images for: Analysis of N6-Methyladenosine Modification Patterns and Tumor Immune Microenvironment in Pancreatic Adenocarcinoma
Source: Front Genet. 2022 Jan 3;12:752025. doi: 10.3389/fgene.2021.752025 (PMC8762218; doi:10.3389/fgene.2021.752025)

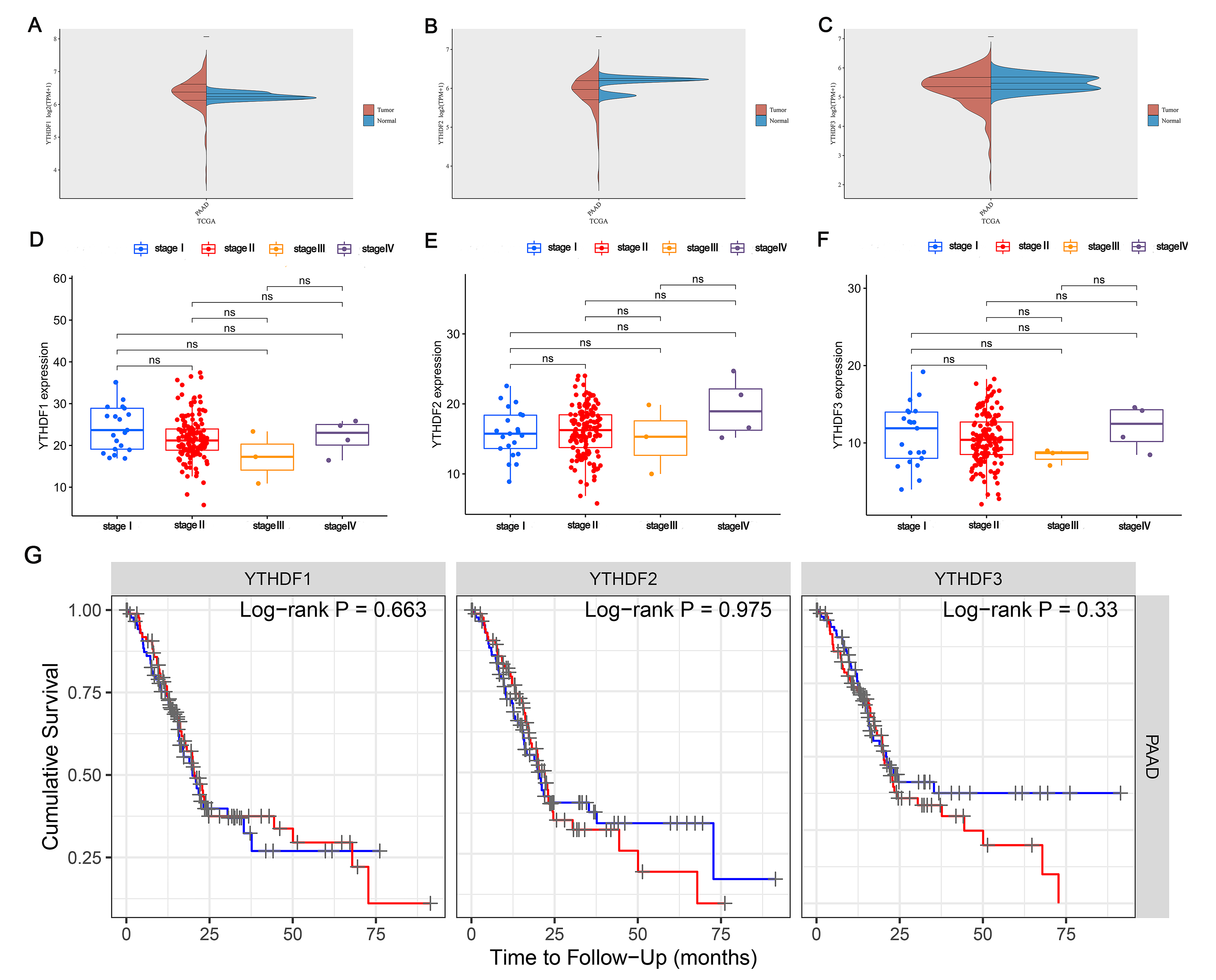

Supplement: Supplementary file 2 [file Image3.TIF]

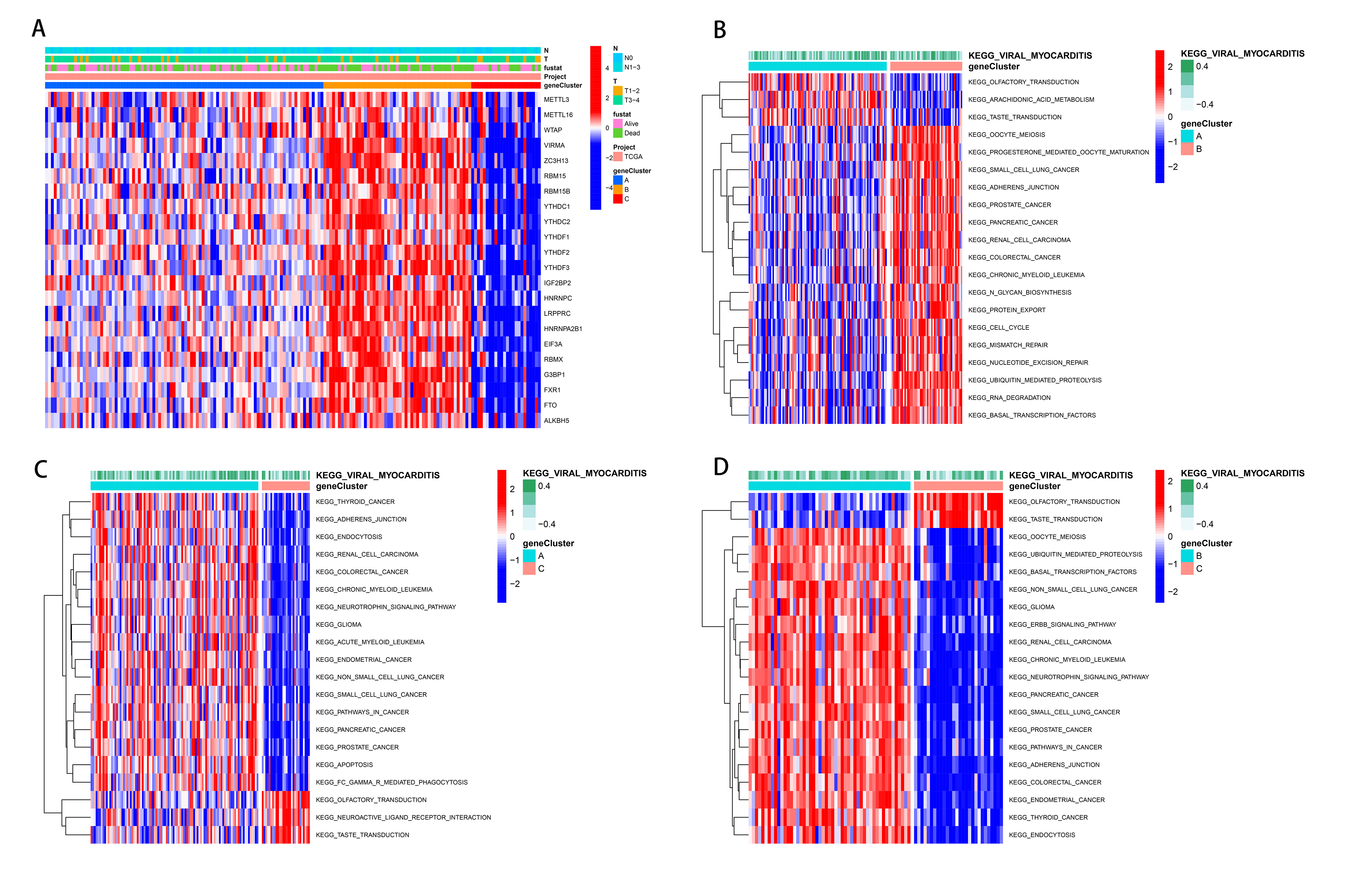

Supplement: Supplementary file 3 [file Image2.TIF]

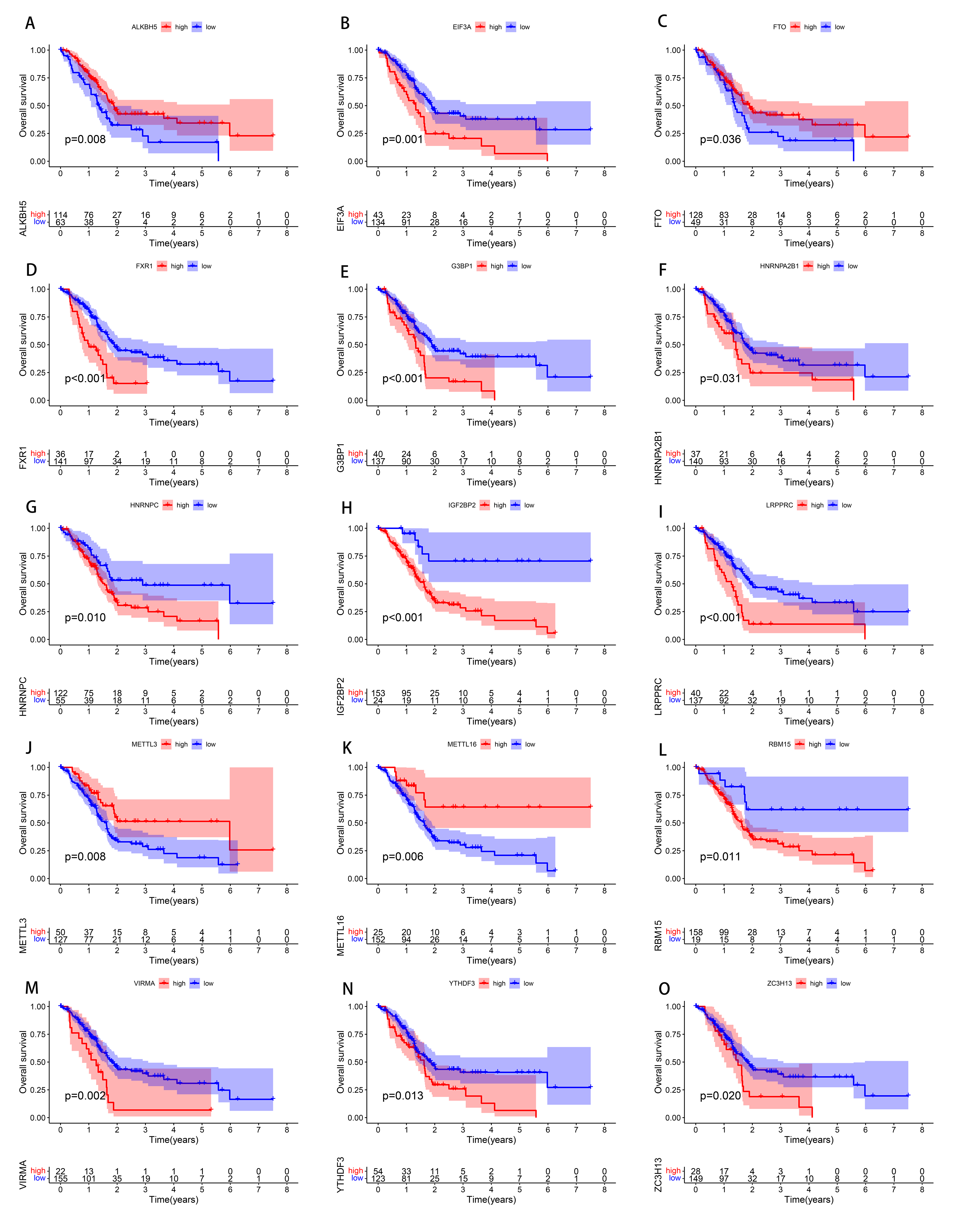

Supplement: Supplementary file 4 [file Image1.TIF]
