# Supplementary material for: Analysis of N6-Methyladenosine Modification Patterns and Tumor Immune Microenvironment in Pancreatic Adenocarcinoma
Source: Front Genet. 2022 Jan 3;12:752025. doi: 10.3389/fgene.2021.752025 (PMC8762218; doi:10.3389/fgene.2021.752025)
Supplement: Supplementary file 5 [file Table2.DOCX]

Table S2 prognostic-related genes between DEGs.

| ID | HR | HR.95L | HR.95H | pvalue |
| --- | --- | --- | --- | --- |
| IPO7 | 1.908846 | 1.323394 | 2.753294 | 0.000542 |
| SMC4 | 1.847169 | 1.345693 | 2.53552 | 0.000146 |
| PAK2 | 1.928675 | 1.310606 | 2.838219 | 0.000862 |
| IQGAP1 | 1.828514 | 1.30852 | 2.55515 | 0.000408 |
| KPNA4 | 2.53872 | 1.58416 | 4.068464 | 0.000108 |
| STRN | 2.002332 | 1.328872 | 3.017096 | 0.000903 |
| NRAS | 1.936465 | 1.345266 | 2.787476 | 0.000377 |
| GMPS | 2.537165 | 1.562345 | 4.120221 | 0.000167 |
| NMD3 | 2.539648 | 1.585409 | 4.068231 | 0.000106 |
| ADAM10 | 1.719285 | 1.259229 | 2.347419 | 0.000648 |
| KIF20B | 1.932416 | 1.442013 | 2.589596 | 1.03E-05 |
| COMMD2 | 2.141478 | 1.366898 | 3.35499 | 0.000886 |
| SP1 | 2.003797 | 1.358914 | 2.954714 | 0.000452 |
| TMOD3 | 2.041107 | 1.437373 | 2.898424 | 6.67E-05 |
| RAB10 | 2.252855 | 1.406835 | 3.60764 | 0.000723 |
| PPFIBP1 | 1.808268 | 1.313071 | 2.490218 | 0.000285 |
| ACTL6A | 2.867798 | 1.723742 | 4.771171 | 4.98E-05 |
| EIF2A | 2.641786 | 1.591411 | 4.385437 | 0.000172 |
| PALB2 | 2.00754 | 1.355723 | 2.972743 | 0.000503 |
| AHNAK | 1.69483 | 1.259541 | 2.280553 | 0.000495 |
| INCENP | 2.058391 | 1.440252 | 2.941828 | 7.42E-05 |
| PDCD10 | 2.322664 | 1.513037 | 3.565522 | 0.000116 |
| ZDHHC5 | 2.431773 | 1.44432 | 4.094329 | 0.000829 |
| LSM12 | 2.553257 | 1.502972 | 4.337488 | 0.000526 |
| CNBP | 2.959058 | 1.592392 | 5.498659 | 0.0006 |
| PIK3CB | 1.986203 | 1.367649 | 2.884513 | 0.000313 |
| FNDC3B | 1.797629 | 1.333613 | 2.423095 | 0.000118 |
| GNAI3 | 2.098699 | 1.35514 | 3.250248 | 0.000895 |
| SGMS2 | 1.460635 | 1.174638 | 1.816265 | 0.000655 |
| VPS26A | 2.253386 | 1.416239 | 3.585376 | 0.000607 |
| STK3 | 2.22506 | 1.468797 | 3.370712 | 0.000161 |
| NIP7 | 2.170342 | 1.411412 | 3.337355 | 0.000416 |
| ERGIC2 | 2.203612 | 1.441058 | 3.36968 | 0.000266 |
| ATL3 | 1.787394 | 1.27935 | 2.497188 | 0.000664 |
| SLC5A3 | 1.533625 | 1.195481 | 1.967414 | 0.000766 |
| DPYD | 1.553712 | 1.207106 | 1.999841 | 0.000623 |
| PTPN14 | 1.68879 | 1.259524 | 2.264358 | 0.000462 |
| PRIM2 | 2.179318 | 1.3887 | 3.420054 | 0.000704 |
| EPS8 | 1.783839 | 1.364447 | 2.332142 | 2.31E-05 |
| LMNB1 | 1.638744 | 1.258484 | 2.133902 | 0.000246 |
| SAV1 | 1.906546 | 1.331356 | 2.730238 | 0.000428 |
| ANO6 | 1.788481 | 1.298598 | 2.463167 | 0.000371 |
| B3GNT5 | 1.665861 | 1.292095 | 2.147748 | 8.26E-05 |
| PRKCI | 1.757073 | 1.334325 | 2.31376 | 5.97E-05 |
| RBBP8 | 1.812166 | 1.302442 | 2.521377 | 0.000419 |
| CLSPN | 1.796411 | 1.314511 | 2.454974 | 0.000237 |
| MAP3K13 | 2.335669 | 1.456729 | 3.744932 | 0.000429 |
| STK38L | 1.643168 | 1.247973 | 2.163509 | 0.000403 |
| JAG1 | 1.60671 | 1.240357 | 2.08127 | 0.000329 |
| IL1RAP | 1.596638 | 1.248252 | 2.042258 | 0.000195 |
| ARHGAP18 | 1.815619 | 1.319043 | 2.499138 | 0.000254 |
| MRPS22 | 3.278009 | 1.857727 | 5.784136 | 4.18E-05 |
| SSR3 | 2.075929 | 1.414933 | 3.045713 | 0.000188 |

Abbreviations: DEGs: differentially expressed genes; HR: hazard ratio.
